# Institutional Notes and News

**Published:** 1910-10-08

**Authors:** 


### Institutional Notes and News.

**Hornsey Cottage Hospital**

The Council of the Hornsey Cottage Hospital met recently to consider, at the suggestion of the chairman, Mr. J. Scott Balfour, the medical committee's statement concerning a case of alleged carelessness on the part of the hospital's staff. The case was that of a child received on July 11, who was operated upon the day following, but who died owing to complications on the 15th. A rumour started that death was due to neglect on the part of the nurses, and so an inquiry was held on July 26. After evidence, including that of the child's father, had been heard, and after the committee had sat for three hours, the charge against the nurses was held to be unfounded. The father was then recalled, and, on being asked whether he was satisfied with the finding of the committee, replied that he was. This should have closed the affair. Apparently however gossip still continued, and no doubt it was to show on what an unfair basis it rested that Mr. Scott Balfour asked the chairman of the Medical Committee to make a statement the other day. His action in doing so was a straightforward meeting of a most elusive attack. We feel it due to the hospital that the facts should be stated.

**Ilkeston Hospital.**

The Ilkeston Hospital has now started on its second quarter of a century, and the twenty-sixth report shows that its usefulness and efficiency continue to grow. The ward cases for the past year numbered 107, while 305 persons were treated in the outpatient department. It is satisfactory to observe that the income for the past year has created a record, reaching a total of £1,142, while during the same period the expenses of maintenance have been reduced slightly. This gives a balance-sheet likely to impress the public with the idea that money subscribed to the Ilkeston Hospital is money spent carefully; indeed, the fact that electric light has been installed without altering the above-mentioned economy is a good illustration of what good management can do. Mr. Seth Manners, the Chairman wisely pointed out, however, that the small number of private subscribers was an unfortunate sign, even though the subscriptions of employers remained undiminished. The Ilkeston Fair, which was extended for the benefit of the hospital, and produced £86, we presume, must have been of little benefit to anybody, as the authorities have said that its extension cannot be repeated. Whatever the reason in this case, it is broadly true that fairs survive nowadays but hardly. The hospital has now a reserve fund amounting to £2,561.

**Newcastle Royal Victoria Infirmary.**

Sir George Hare Philipson presided at a special meeting of the Newcastle Royal Victoria Infirmary House Committee, held recently to consider the instructions sent to it by the governors to reopen St. Luke's Chapel at the infirmary and to provide "facilities for Anglican, Free Church, and such other services as may be required in the interests of the patients and staff." After some discussion the following resolution was agreed to: "That in face of the opinion of Mr. W. W. Gibson, hon. solicitor to the institution, to the effect that any governor could easily get an injunction against the house committee should they carry out the instructions of the last quarterly Court, and that the cost of such injunction would have to be paid by the members of the house committee personally, and further that any refusal of members of the house committee to pay such costs, or, in case of the house committee refusing to obey the injunction, the members may be imprisoned for contempt of court, we decide (1) that we cannot carry out the order of the last quarterly Court; (2) that we report this decision to a special court of governors to be held on Saturday, October 22." We sympathise deeply with the governors for being forced into this invidious dilemma. It is to be feared that the motives of those recalcitrants who have fomented the difficulty are not in accord with the principles of either party. It is their duty to keep the hospital out of the dispute, which is outside its scope altogether, by meeting together and deciding on some solution to be abided by faithfully, which they should then present to the governors for adoption. In this way only can the disputants prove their good faith. To throw the burden of decision on the hospital is unjust and unpardonable.

**Children's Sanatorium, Holt.**

The Children's Sanatorium for Consumptives, Holt, Norfolk, which issued an appeal in July, backed by Earl Cawdor, Sir Edgar Speyer, and others, has received a promise from Mr. Otto Beit of £5,000. This will enable the committee to proceed with the building without any further delay, and the committee hope that intending subscribers to the building fund will divert their gifts to the maintenance fund, by the help of which alone the buildings can be made available. It is perhaps necessary to emphasise again the importance of this point, as the public has been slow to see that hospitals and sanatoria not only have to be built but to be kept going, and a fine building without a fine endowment becomes an incubus instead of a blessing. Having received, as already noted in these columns, the support of King Edward's Hospital Fund, we cannot think that Mr. Beit's generous promise will lack other supporters to give to the children the full benefit of the scheme. The hon. secretary, 68 Denison House, Vauxhall Bridge Road, S.W., will be glad to receive all communications.

**St. George's Hospital.**

Dr. S. Squire Sprigge, the editor of the *Lancet*, distributed the prizes at St. George's Hospital Medical School on Saturday, which thus was, we believe, the first to open the medical session. Dr. Sprigge pointed out in his address that medical education did not end with registration or qualification. Thus the two contrary views as to the value of examinations and prizes became at once reconcilable. Examinations were a means to the end and no more; prizes were an incentive and no more. He protested against the multiplicity of the examinations and the intricacy which of late had marked the medical curriculum. The story of pathological progress during the last twenty years might be summed up in the statement that scientific research of the highest kind in physics and chemistry had been made to play a part in practical medicine, therapeutical and preventive. Returning to the question of prizes, Dr. Sprigge said that although the profession of medicine was showing in its workaday proceedings how the finest flowers of its philosophy could be used, the fact remained that prizes came no more in medicine than in other walks of life to the actual exponents of that philosophy. Teaching should be more widely endowed. Into the hands of the leaders of medical thought fell the duty of teaching students, but in London in particular that duty was largely discharged as a gratuitous one.

The shortsightedness and false economy, concluded Dr. Sprigge, of encouraging students freely with exhibitions and scholarships while making scanty provision for those who teach them is simply an exhibition of the old and narrow view of education. It is a wrong policy which leads wealthy and generous persons so much more frequently to found scholarships for the students than lectureships, readerships, fellowships, and professorial chairs for their teachers. Both are wanted, but what right have we to expect that the highly endowed student will receive adequate instruction if we fail to endow his masters? Mr. Clinton Dent, chairman of the Medical School Committee, presided, and those present included Sir William Bennett, Sir Edgcombe Venning, Mr. Robertson Turner, Mr. Brudenell Carter, Mr. Pickering Pick, and Dr. Cyril Ogle.

**Middlesex Hospital School Dinner.**

The annual dinner of the Middlesex Hospital Medical School, held last week, was a great success. Mr. T. H. Kellock presided over a distinguished company, which included Lord Duncannon, M.P., Dr. L. Barlow, Mr. J. Bland Sutton, Dr. Sidney Coupland, Dr. J. K. Fowler, Mr. A. Pearce Gould, Mr. F. Clare Melhado, Sir Henry Morris, Dr. H. Campbell Thomson, Mr. B. S. Simmonds, and Mr. Andrew Clark. The toast of the evening was, of course, the Middlesex Hospital and Medical School, which was proposed by Mr. Kellock, who said : "The appointment of Mr. Andrew Clark as hon. surgeon to the King is the first recognition his Majesty has made of the medical side of the Territorial Force." This was received with great applause, as also was Mr. Kellock's reminder that the Middlesex Medical School was unique among similar London bodies in the fact that it was practically self-supporting.

**Old Students at Bart's.**

The old students' dinner of St. Bartholomew's Hospital and College took place last week, Mr. C. B. Lockwood presiding. His speech revealed the encouraging facts that the number of men coming from the older Universities was increasing, and that the entry of students was full. Mr. Lockwood remarked also on the need for a nurses' home and a large surgical block which is pressing on the hospital, and gave handsome praise to the good work which the new clinical block has performed already. By this block unrivalled facilities are obtainable, which are being made use of to the full. Lord Sandhurst, Dr. Hill (Vice-Chancellor of the University of London), Sir Clifford Allbutt, Mr. Henry Butlin, Sir William Church, Dr. Norman Moore, Mr. Bruce Clarke, Sir Francis Champneys, Sir Dyce Duckworth, Professor Howard Marsh, Dr. Herringham, Sir Lauder Brunton, Mr. D'Arcy Power, and Mr. H. J. Waring were also present.

**St. Thomas's Hospital.**

A very interesting record of hospital progress was given by Mr. Wainwright, who responded to the toast of "St. Thomas' Hospital and Medical School" at the annual dinner of old students last Tuesday. He reminded his listeners that the last twenty years had seen the extinction of a debt of £40,000, the building and equipment of two children's wards, and the filling of five wards which had been previously empty. In one department alone improvement was not manifested, every section of the hospital had received amplification, except the out-patient department, which stood now as it had done in 1871. To do this £40,000 was required, which presented a serious difficulty in view of the scarcity of legacies from which the hospital was suffering, and of the action of the King's Fund, which "absolutely ignored" the special need and work of St. Thomas's. Mr. W. F. Haslam presided at the dinner, at which many distinguished persons were present, among whom we may note Mr. H. T. Butlin, Sir James Porter (Director-General of the Medical Department of the Navy), Sir A. Keogh, Mr. H. G. Turney (the Dean of the Medical School), Sir T. B. Crosby, and Sir T. Chavasse.

